# Xanthan Gum-Stabilized Sunflower Oil Body Emulsions for β-Carotene Delivery: Preparation, Stability, and Digestion Behavior

**DOI:** 10.3390/foods15030567

**Published:** 2026-02-05

**Authors:** Xuan Sheng, Farah Zaaboul, Lixia Chen, Lele Chen, Ruizhi Yang, Luping Zhao

**Affiliations:** 1 Engineering and Technology Center for Grain Processing of Shandong Province, Key Laboratory of Food Processing Technology and Quality Control in Shandong Province, College of Food Science and Engineering, Shandong Agricultural University, 61 Daizong Avenue, Tai’an 271018, China; 2 Laboratory of Biotechnology, Environment, Agri-Food and Health, Faculty of Sciences Dhar El Mahraz, Sidi Mohamed Ben Abdellah University, Fez 30003, Morocco; 3 School of Food and Pharmacy, Zhejiang Ocean University, Zhoushan 316022, China

**Keywords:** sunflower oil bodies, xanthan gum, β-carotene, encapsulation efficiency, gastrointestinal digestion

## Abstract

In this study, we investigated the encapsulation of β-carotene (β-CE) within sunflower oil body (SFOB) emulsions and examined the role of xanthan gum (XG) in enhancing stability and digestion behavior. The optimal conditions were heating at 45 °C for 15 min, ultrasonic treatment at 270 W for 20 min, and homogenization at 80 MPa, achieving encapsulation efficiency (EE) up to 92%. Furthermore, XG was incorporated to improve structural, oxidative, thermal, and digestive stability. More than 1.5% XG enhanced absolute value of zeta potential (21.3 mV to 23.7 mV), reduced particle size (6.52 μm), and prevented phase separation. XG-coated emulsions exhibited improved stability under heating and oxidative conditions. Additionally, XG enhanced protein digestibility and lipid hydrolysis, as well as the bioaccessibility of β-CE during gastrointestinal digestion. The XG coating also improved photostability under sunlight and UV exposure, with 2% XG emulsions showing the least degradation of β-CE. Moreover, the 2% XG emulsion demonstrated the highest release of free fatty acids (85.75%) and β-CE utilization (80%). These results highlight the potential of SFOB-XG emulsions for the effective delivery of lipophilic bioactives.

## 1. Introduction

β-carotene (β-CE) is a highly valued lipophilic compound widely recognized for its role as a precursor of vitamin A and its potent antioxidant activity [1,2]. It contributes significantly to human health by supporting vision, immune function, and cellular protection against oxidative stress [3]. Due to these functions, β-CE is commonly used in the development of functional foods and nutraceuticals. However, its application in food systems is severely limited by several physicochemical challenges, including poor water solubility [4], chemical instability (particularly in the presence of light, heat, and oxygen) [4], and low bioavailability during digestion [5]. These limitations make it difficult to incorporate into aqueous-based formulations and reduce its efficacy as a nutritional additive. To overcome these barriers, various delivery systems have been developed, with emulsions being among the most widely used strategies for solubilizing and protecting β-CE and other lipophilic bioactives [6,7,8].

Oil-in-water emulsions provide an effective way to disperse hydrophobic compounds in water-based food matrices, enhancing their stability and absorption [9]. However, conventional emulsions often rely on synthetic surfactants or highly processed emulsifiers, which can present problems such as toxicity, poor biocompatibility, and consumer preference for clean-label, natural ingredients. This has led to growing interest in natural emulsions from plant materials, which are generally recognized as safe and more compatible with food systems [10]. Among these are oil bodies (OBs), also called oleosomes, which are considered promising candidates for the encapsulation and delivery of lipophilic compounds such as β-CE [11]. OBs are natural lipid storage organelles found in seeds and nuts, consisting of a triacylglycerol (TAG) core surrounded by a monolayer of phospholipid embedded with structural proteins [12]. This structure protects the internal lipid core from enzymatic degradation and oxidation while also providing emulsifying functionality [13]. During aqueous extraction, OBs are often accompanied by storage proteins, also referred to as exogenous proteins, some of which become adsorbed at the OB interface. Depending on the treatment applied to OBs, these extrinsic proteins can enhance interfacial stability by acting as a second coat on oil bodies [14]. This system enables OBs to function as both the emulsifier and the oil phase, creating an all-in-one, food-grade delivery vehicle.

An increasing number of studies have explored the use of OBs to encapsulate functional lipophilic compounds. For example, peanut oil bodies were used to encapsulate and deliver curcumin with enhanced bioaccessibility [15]. More recently, a study successfully encapsulated β-CE using flaxseed OBs in a solvent-free process, demonstrating the potential of OBs as a promising candidate for encapsulation and delivery [11]. These findings highlight the versatility and biocompatibility of OB-based systems in improving the functionality of poorly soluble compounds. In addition, sunflower oil body (SFOB) is rich in linoleic acid and tocopherol (392 mg/kg OBs, dry basis), which are beneficial for heart health [16], making SFOBs a promising prospect for use in food-grade delivery vehicles.

Despite the intrinsic stability of OB emulsions, additional structural stabilization may be necessary for practical food applications, especially under heat, acid, or ionic stress. Polysaccharides such as xanthan gum (XG) have been widely used to enhance emulsion stability by increasing viscosity, providing steric hindrance, and reducing droplet aggregation [17,18]. XG is an anionic, food-grade biopolymer that remains stable under a wide range of environmental conditions and is compatible with clean-label formulations. Xanthan gum can also reduce blood glucose and total cholesterol levels in patients with diabetes [19].

Upon ingestion, β-CE must be released from the food matrix, solubilized into mixed micelles, and absorbed by the intestinal mucosa to exert its nutritional effects. These steps, collectively termed bioaccessibility, are therefore essential factors determining its nutritional efficacy.

Based on the fact that OB contains the oil phase and natural emulsifiers (phospholipids and oleosins), it has the potential to encapsulate lipid-soluble β-CE. To incorporate β-CE into the oil phase of SFOB, the effects of heating (30–45 °C, 5–45 min), ultrasonication (90–330 W, 10–60 min), and homogenization (20–110 MPa) on encapsulation efficiency, size, and zeta potential were analyzed. Then, we formulated β-CE-loaded SFOB emulsions with and without XG. We assessed their physicochemical properties and stability under various environmental stresses over different storage periods. To evaluate the potential of our SFOB emulsion to enhance β-CE bioaccessibility, the release and bioaccessibility of β-CE during simulated in vitro gastrointestinal digestion were evaluated.

In this context, the present study aimed to develop a β-CE delivery system based on sunflower oil bodies and XG, targeting improved encapsulation efficiency, thermal and oxidative stability of the emulsion, light stability, and bioaccessibility of β-CE. While sunflower oil bodies have been studied for encapsulation, their use in combination with XG to create a multifunctional delivery system for β-CE has not been thoroughly investigated. This study provides a comprehensive approach by (1) optimizing the physical encapsulation of β-CE into SFOBs using a combined heat-ultrasonic-homogenization method, (2) systematically evaluating how XG concentration affects not only physical stability (against heat, light, and oxidation) but also interfacial properties, and (3) directly linking this stabilization to improved gastrointestinal fate and β-CE bioaccessibility. This integrated analysis offers new insights into designing clean-label, OB-based delivery systems with enhanced functional performance.

## 2. Materials and Methods

### 2.1. Materials

The sunflower seeds (Jinkui No. 5) were bought at a local market. β-CE (purity 99%) and food-grade XG (purity 98%) were purchased from Beijing Solarbo Technology Co., Ltd. (Beijing, China). Pancreatin and pepsin were bought from Sinopharm Chemical Reagent Co., Ltd. (Shanghai, China). Other reagents were purchased from Tianjin Kaitong Chemical Reagent Co., Ltd. (Tianjin, China). All reagents were analytically pure.

### 2.2. Extraction of SFOB

The extraction method for sunflower seed oil body (SFOB) was adapted from the method of [20]. Sunflower seeds were soaked in deionized water at 4 °C for 18 h. The soaked seeds were mixed with fresh deionized water at a ratio of 1:8 and then ground for 2 min. The mixture was filtered through three layers of defatted gauze, and the resulting slurry was collected. The slurry was adjusted to pH 9.5 and centrifuged at 7000× *g*, 4 °C, for 30 min. The upper layer was collected, dispersed again in deionized water, and centrifuged under the same pH and centrifugation conditions to collect the upper cream. The cream was SFOB, with a water content of 22.29%, protein (dry basis) of 2.84%, and lipid content (dry basis) of 94.42%.

### 2.3. Preparation of Emulsion

The 10% SFOB emulsions (pH 7.0) were prepared by mixing 10.0 g SFOB with 90.0 g deionized water, and β-CE (0.5%, *w*/*w*) was added to the emulsions. The mixtures were stirred at 30–45 °C (β-CE was decomposed at ≥50 °C) in a water bath for 5–45 min. After heating, ultrasonication (90–330 W, 10–60 min; FS Ultrasonic Processor, Shanghai Shengxie Ultrasonic Instrument Co., Ltd., Shanghai, China) and high-pressure homogenization (20–110 MPa; APV-2000, APV Co., Ltd., Berlin, Germany) were successively performed to obtain various emulsions containing β-CE (named SFOB-β-CE). Finally, XG (0–2.0%, *w*/*w*) was added to SFOB-β-CE to obtain XG-SFOB-β-CE emulsions at room temperature, which were stirred for 10 min. The schematic diagram illustrating the emulsion preparation process is provided in Supplementary Figure S1.

### 2.4. Particle Size and Zeta Potential Determination

All emulsions were diluted 400-fold with 20 mmol/L phosphate-buffered solution (PBS, pH 7.0). Particle sizes were measured using a Laser Particle Size Analyzer (Beijing Haixinrui Technology Co., Ltd., Beijing, China) at 25 °C. Zeta potentials were determined with the Nano-ZS Laser Particle Size Analyzer (Malvern Instruments Ltd., London, UK) at 25 °C. Particle size data were reported as D4,3.

### 2.5. Determination of the Encapsulation Efficiency and Retention Rate of β-CE

#### 2.5.1. Encapsulation Efficiency of β-CE

The encapsulation efficiency (EE) of β-CE was measured [21]. The absorbance of n-hexane/ethanol (2/1, *v*/*v*) solutions containing β-CE (0, 1, 2, 3, 4, 5 μg/mL) was measured at 450 nm. The n-hexane/ethanol (2/1, *v*/*v*) solution was used as the blank to prepare the standard curve (Figure S2). One milliliter of fresh SFOB-β-CE was mixed with 4 mL of n-hexane/ethanol (2/1, *v*/*v*) for 30 s and centrifuged at 6300× *g* for 5 min. The supernatant and the lower layer emulsion were collected. The absorbance of the supernatant was measured at 450 nm. For the lower layer emulsion, 4 mL ethanol and 8 mL n-hexane were added, then vortexed 3 times for 15 min. After centrifugation at 6300× *g* for 5 min, the supernatant was collected, and its absorbance was measured at 450 nm. The β-CE contents (M_0_, M_1_) were calculated using the standard curve (Figure S2). The calculation formula for EE is:

$$\mathrm{EE}\;\left(\%\right)\;=\;\frac{M_{1}}{M_{0}\;+\;M_{1}}\;\times \;100$$

where M_0_ is unembedded β-CE concentration in the aqueous phase, mg/mL; M_1_ is the concentration of embedded β-CE, mg/mL.

#### 2.5.2. Retention Rate of β-CE

Fresh SFOB-β-CE was stored in the dark for 7 days at 4 °C. The stored SFOB-β-CE (1 mL) was mixed with 12 mL n-hexane/ethanol (2/1, *v*/*v*) for 15 min and centrifuged at 6300× *g* for 5 min. The absorbance of the collected supernatant was measured at 450 nm. The β-CE contents (C_0_, C) were calculated using the standard curve (Figure S2). The calculation formula for retention rate is:

$$\mathrm{retention}\;\mathrm{rate}\;(\%)\;=\;\frac{C}{C_{0}}\;\times \;100$$

where C is the content of β-CE after storage for a certain time, mg/mL; C_0_ is the initial content of β-CE, mg/mL.

### 2.6. Characterization of the Stability of XG-SFOB-β-CE Emulsion

#### 2.6.1. Storage Stability

The XG-SFOB-β-CE was stored in the dark for 0, 1, 3, 5, and 7 days at 4 °C. The zeta potential, particle size, visual observation, and retention rate of β-CE were evaluated.

#### 2.6.2. Thermal Stability

Thermal stability was assessed according to Tian et al. [21] with some modifications. XG-SFOB-β-CE was heated in a water bath at 100 °C for 15 min, and the emulsion without XG served as the control. Zeta potential, particle size, and visual observations were measured.

#### 2.6.3. Oxidative Stability

The XG-SFOB-β-CEs emulsions were stored in the dark for 0, 1, 3, 5, 9, and 14 days at 37 °C.

The peroxide value (POV) was determined according to the method of Shen et al. [22] with slight modifications. A 0.3 mL sample of emulsion was added to 1.5 mL of isooctane–isopropanol solution (*v*:*v* = 2:1). The mixtures were centrifuged at 1500× *g* for 10 min. Then, 0.2 mL of the supernatant was mixed with 20 μL of 3.94 mol/L KSCN and 20 μL of 0.072 mol/L Fe^2+^ solution, and the volume was adjusted to 10 mL with methanol–n-butanol solution (*v*:*v* = 2:1). The reaction was carried out for 20 min in the dark. Absorbance was measured at 510 nm using a UV-5100 spectrophotometer (Shanghai Yuan Analysis Instrument Co., Ltd., Shanghai, China). The POV was calculated according to the standard curve (Figure S3) using the following formula:

$$\mathrm{POV}\;\left(\mathrm{mmol}/\mathrm{kg}\right)\;=\frac{{M\;\times \;A}_{510}}{55.84\;\times \;2\;\times \;m_{0}}$$

where A_510_ is the absorbance value at 510 nm; M is the slope of the Fe^3+^ standard curve; and m_0_ is the colorimetric equivalent sample mass in g.

The secondary products of lipid oxidation were evaluated using thiobarbituric acid reactive substances (TBARS) according to the method of Shen et al. [22]. First, TBA (187.5 mg) and trichloroacetic acid (7.5 g) were dissolved in 50 mL of 0.25 mol/L HCl. Then, 0.5 mL of sample was mixed with 1 mL of TBA solution in a test tube and placed in a boiling water bath for 15 min. Afterward, 0.3 mL of trichloromethane was added, and the mixture was centrifuged at 2000× *g* for 15 min. The absorbance of the supernatant was measured at 532 nm. A standard curve prepared with 1,3,3-tetramethoxypropane (0–30 μmol/L) was used to calculate the TBARS content.

### 2.7. Rheological Properties

Rheological properties were determined using a rheometer (Anton Paar Co., Ltd., Vienna, Austria) according to Zhao et al. [23]. A 50 mm aluminum plate clamp and a spacing of 1 mm were selected. The measurement temperature was 25 °C, the shear rate was 0.1–100 s^−1^, and the apparent viscosity was recorded. The model used to fit the flow behavior data was the Ostwald–de Waele model, also known as the power law, and represented as follows:

$$\eta =\;K\times \gamma^{n-1}\;\;$$

where η is the viscosity (Pa·s), γ is the shear rate (s^−1^), K is the consistency index (Pa·s^n^), and *n* is the flow behavior index.

### 2.8. Light Stability of β-CE

The fresh XG-SFOB-β-CE was placed in a simulated sunlight incubator with a 45 W (2000 lux) sunlight lamp at 25 °C. Samples were collected after 1, 3, and 7 days to determine the β-CE content in the emulsions and calculate the retention rate of β-CE.

The freshly prepared XG-SFOB-β-CE was irradiated with ultraviolet light (365 nm) for 4, 8, 12, 24, and 48 h under a 36 W UV lamp, with the distance between the UV lamp and the emulsion surface approximately 5 cm (120 µW/cm^2^). The retention rate of β-CE in the emulsion was calculated. β-CE was dissolved in sunflower seed oil (designated FS-oil, 0.5% *w*/*w*) to serve as the control.

### 2.9. In Vitro Gastrointestinal Digestion

The in vitro digestion model followed the method described by He et al. [24]. Simulated gastric fluid (SGF) was prepared with 0.43 mol/L NaCl, and 80.0 g of coated and uncoated SFOB emulsion was mixed with 20 mL of SGF. The pH of the mixture was adjusted to 4.0 using HCl (0.2–2 mol/L), after which pepsin was added at a final concentration of 0.68 mg/mL. Gastric digestion was carried out at 37 °C for 2 h. After this phase, the pH was raised to 7.0 using KOH (0.2–2 mol/L) to initiate the intestinal digestion stage. Simulated intestinal fluid (SIF) was composed of 0.75 mol/L NaCl, 25 mmol/L KCl, and 0.3 mol/L CaCl_2_. Bile salts were added to 57.6 mL of SIF to reach a final concentration of 10.8 mg/mL, followed by the addition of pancreatin at 6.35 mg/mL. This intestinal phase was conducted at 37 °C and pH 7.0 for 3 h. The amount of KOH consumed during the reaction was continuously monitored using a pH-STAT automatic potentiometric titration system, and the release of free fatty acids was calculated accordingly.

### 2.10. The Release of Free Fatty Acid

The release of FFA was calculated based on the consumption of 0.1 mol/L KOH solution during the intestinal phase [25], using the following equation:

$$\mathrm{FFA}\;(\%)\;=\;\frac{V_{KOH}\times C_{KOH}\times M}{M_{lipid}\times 2}\;\times \;100$$

where V_KOH_ (L) and C_KOH_ (mol/L) represent the volume and concentration of KOH consumed, respectively; M is the molecular weight of sunflower oil, 876.56 g/mol; and M_lipid_ is the weight of TAGs in SFOB (g).

### 2.11. The Bioaccessibility of β-CE

The bioaccessibility of β-CE after digestion was measured according to the method described by Fu et al. [26]. It was defined as the fraction of β-CE released from the food matrix and solubilized in the mixed micelle phase after digestion. Specifically, after the intestinal phase, a 15 mL mixture was centrifuged at 7000× *g* and 4 °C for 30 min. After centrifugation, the clear middle layer, representing the micelle phase containing solubilized β-CE, was collected for measurement at 450 nm to determine β-CE concentration. This was defined as the fraction released from the food matrix and solubilized in the mixed micelle phase after digestion:

$$\mathrm{Bioaccessibility}\;\beta \text{-}\mathrm{CE}\;\left(\%\right)\;=\frac{C_{Micelle}}{C_{Initial}}\;\times \;100$$

where C_Micelle_ is the β-CE concentration in the micelle fraction and C_Initial_ is the β-CE concentration in the initial emulsion.

### 2.12. Statistical Analysis

All analyses were performed in triplicate, and the data are expressed as mean ± standard deviation. Data charts were created using professional drawing software (Origin 9.1), and statistical analysis was conducted using SPSS 24. Mean values of different treatments were compared at the 5% significance level.

## 3. Results and Discussion

### 3.1. Effects of Different Treatment Conditions on SFOB Emulsion Encapsulating β-Carotene

As shown in Table 1, heat treatment, ultrasonic treatment and homogenization have significant effects (*p* < 0.05) on the zeta potential, particle size and β-CE encapsulation efficiency (EE) of the SFOB-β-CE emulsions. These effects were determined using a one-factor-at-a-time approach, where each sample underwent the complete sequence of heating, ultrasonication, and homogenization, with only the parameter under investigation being varied (see Table 1 for fixed baseline conditions).

Experimental Design Note: All emulsions were prepared using the full sequential process (heating → ultrasonication → homogenization). When one parameter was tested, the others were fixed as follows:

• Temperature test (30–45 °C): 15 min heating, 330 W ultrasonication, 80 MPa homogenization.
• Heating time test (5–45 min): 40 °C, 330 W ultrasonication, 80 MPa homogenization.
• Ultrasonic power test (90–330 W): 45 °C, 15 min heating, 80 MPa homogenization.
• Ultrasonication time test (10–60 min): 40 °C, 330 W ultrasonication, 80 MPa homogenization.
• Homogenization pressure test (20–110 MPa): 40 °C, 30 min heating, 330 W ultrasonication.

Heating temperature significantly influenced the encapsulation efficiency (EE), zeta potential, and particle size of SFOB emulsions carrying β-CE. As the temperature increased from 30 °C to 45 °C, EE increased sharply from 45.6 ± 0.5% to 92.1 ± 0.6% (*p* < 0.05). This enhancement is attributed to the partial thermal unfolding of interfacial proteins such as oleosins, which improves the permeability of the OB membrane for β-CE incorporation [27]. Concurrently, the zeta potential became more negative, with its absolute value increasing from 21.10 ± 0.21 mV to 23.2 ± 0.4 mV, indicating improved electrostatic stabilization due to the release of extrinsic proteins [23,28]. Particle size increased only slightly from 17.1 ± 0.2 μm to 18.2 ± 0.4 μm (*p* < 0.05), suggesting minor interfacial reorganization. Within the tested range, 45 °C was selected as the optimal temperature, balancing high encapsulation with stable emulsion characteristics.

Heating duration was optimized at 45 °C. EE increased from 84.2 ± 0.2% to a peak of 91.9 ± 0.2% at 15 min, then plateaued. Prolonged heating beyond 15 min led to a notable increase in particle size (to 25.3 ± 0.2 μm at 45 min) and a progressively less negative zeta potential, indicating droplet aggregation and loss of interfacial integrity [28]. Hence, 15 min was identified as the optimal heating duration.

Ultrasonic power critically remodeled the emulsion structure. Increasing power to 270 W maximized EE (92.5 ± 0.9%) and produced the smallest particle size (17.8 ± 0.3 μm). The cavitation and shear forces disrupt the oil body interface, facilitating β-CE diffusion into the lipid core and exposing charged protein residues, which increased the absolute zeta potential value from 20.1 mV to 22.8 mV [11]. Thus, 270 W was determined as the optimal ultrasonic power.

The influence of ultrasonic duration was assessed at 270 W. Increasing time from 10 min to 20 min sharply increased EE from 83.9% to 91.1%, with further extension maintaining EE above 90%. This improvement is attributed to the prolonged application of cavitational energy, which ensures complete disruption of protein aggregates, facilitates thorough dissolution of β-CE crystals into the oil phase, and promotes a homogeneous distribution, thereby maximizing the interfacial area available for encapsulation. Particle size decreased correspondingly from 26.0 μm to 17.9 μm, and the zeta potential reached −24.6 mV at 60 min. As improvements stabilized after 40 min, 20 min was selected as the optimum duration to achieve efficient encapsulation without over-processing [11].

Homogenization pressure had a pronounced effect on droplet size. Increasing pressure to 80 MPa maximized EE (90.8 ± 0.6%), produced a strongly negative zeta potential (absolute value: 21.54 ± 0.13 mV), and dramatically reduced particle size to 15.1 ± 0.4 μm. Higher pressure (110 MPa) further reduced size but did not improve EE. Consequently, 80 MPa was selected as the optimal pressure.

Storage stability over 7 days reflected the combined effects of these parameters. All emulsions exhibited a decline in EE and an increase in particle size. However, emulsions prepared under the optimized conditions (45 °C, 15 min; 270 W, 20 min, 80 MPa) showed the best retention of encapsulation efficiency and the smallest size increase, confirming their superior stability. Nevertheless, noticeable deterioration indicated that single-layer SFOB emulsions required additional stabilization.

Based on both initial physicochemical properties and 7-day storage performance, the optimal processing conditions were identified as heating at 45 °C for 15 min, ultrasonic treatment at 270 W for 20 min, and homogenization at 80 MPa. Under these conditions, the maximum encapsulation efficiency reached approximately 92%. Nevertheless, the limited storage stability highlighted the need for additional interfacial protection, which motivated the introduction of a xanthan gum coating in the subsequent section.

### 3.2. The Influence of Xanthan Gum on the Physical Properties and Viscosity of SFOB-β-CE Emulsions

**Figure 1:**
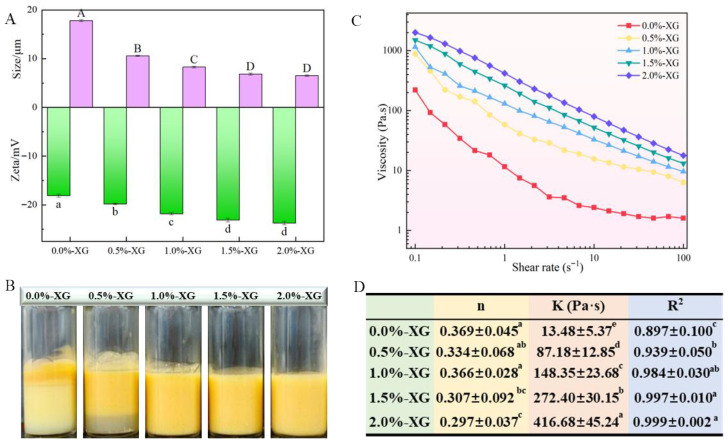
Effects of XG concentrations on zeta potential (**A**), average particle size (**A**), visual observations (**B**), and viscosity behavior (**C**,**D**) of fresh XG-SFOB-β-CE emulsions. Different lowercase and uppercase letters represented significant differences (*p* < 0.05) for the different XG concentrations.

To improve the stability of SFOB-β-CE emulsions, XG was added at various concentrations to form a secondary coating around the SFOBs loaded with β-CE. As shown in Figure 1A, the zeta potential decreased from −18.1 mV (0% XG) to −21.3 mV (1.5% XG)–−23.7 mV (2.0% XG) with increasing XG concentration, indicating that XG increased the negative surface charge of droplets and enhanced electrostatic repulsion between droplets [29]. Particle size also decreased from 17.79 μm (0.0% XG) to 6.52 μm (more than 1.5% XG). In contrast to our results, Hemar et al. found that increasing XG concentration resulted in larger particle size of casein emulsions, which was attributed to the increased viscosity of the emulsion continuous phase and limited the movement of droplets [30]. Zhang et al. found that higher XG concentrations resulted in smaller, more uniform particle distributions of S/O/W (solid/oil/water) emulsions due to increased viscosity and interfacial stabilization [31]. This increased viscosity of the aqueous phase caused by higher XG concentrations led to the formation of a thicker interfacial layer around the droplets. This protective layer reduces droplet collision and aggregation frequency, thereby decreasing the average particle size [32]. Therefore, increased viscosity (Figure 1C,D) and increased negative charge (Figure 1A) might result in the decreasing particle size.

Visual observation of the emulsions (Figure 1B) showed that formulations containing less than 1.0% (*w*/*w*) XG remained unstable and exhibited phase separation. This instability is attributed to insufficient surface charge, which is not strong enough to overcome attractive inter-droplet forces, such as van der Waals interactions. When the XG concentration exceeded 1.0% (*w*/*w*), the XG molecules began to form a dense network through intra- and intermolecular hydrogen bonding, increasing steric hindrance and improving emulsion stability. Furthermore, at these higher concentrations, we propose that XG can directly interact with the SFOB interface. The anionic polysaccharide chains likely adsorb to the oil body surface through electrostatic and polar interactions between their carboxyl groups and positively charged patches (e.g., on lysine or arginine residues) of the interfacial proteins, such as oleosins. This adsorption forms a secondary, protective biopolymer layer around the droplets, a phenomenon observed in other protein-polysaccharide stabilized emulsions [33]. This direct interfacial binding, complementing the bulk viscosity effect, is a crucial mechanism for the enhanced stability, providing a strong electrosteric barrier against droplet coalescence [34]. Consequently, the emulsions became visually homogeneous and showed no signs of phase separation. All these observations are linked to viscosity changes caused by the thickening effect of XG. Therefore, we evaluated how different XG concentrations affect the flow properties of SFOB-β-CE emulsions. As shown in Figure 1C,D, all emulsions exhibited shear-thinning behavior, meaning their viscosity decreased as the shear rate increased [23]. However, the strength of this effect depended on the concentration of XG. To better understand this, we used the power-law model to calculate the flow behavior index (n) and consistency index (K), which quantify how the emulsions flow.

As the XG concentration increased from 0% to 2%, the flow behavior index (n) decreased from 0.369 to 0.297 (*p* < 0.05), indicating that the emulsions became more shear-thinning. More importantly, the consistency index (K) increased dramatically by over 30 times, from 13.48 Pa·sⁿ to 416.68 Pa·sⁿ (*p* < 0.05). This very large increase in K provides strong rheological evidence for the formation of a dense XG network. The pronounced increase in K values between 1.0% and 2.0% XG (from 148.35 to 416.68 Pa·sⁿ) matched the visual stability (Figure 1B), confirming that higher XG concentrations effectively prevented droplet aggregation. These values support the viscosity curves in Figure 1C; emulsions with 1.0% to 2.0% XG started with high viscosity that dropped quickly as shear rate increased, showing strong shear-thinning behavior. This occurs because the weak gel-like network formed by XG breaks down under shear. In contrast, emulsions with less than 0.5% XG had lower initial viscosity and a more gradual decrease with shear, consistent with their higher n and lower K values. As expected, the emulsion without any XG had the lowest viscosity across the entire shear range, confirming the thickening and stabilizing role of XG. This increase in viscosity is mainly due to XG’s ability to bind water and form a loose network, which restricts droplet movement and improves stability [35].

### 3.3. The Influence of Xanthan Gum on the Storage Stability of SFOB–β-CE Emulsion

**Figure 2:**
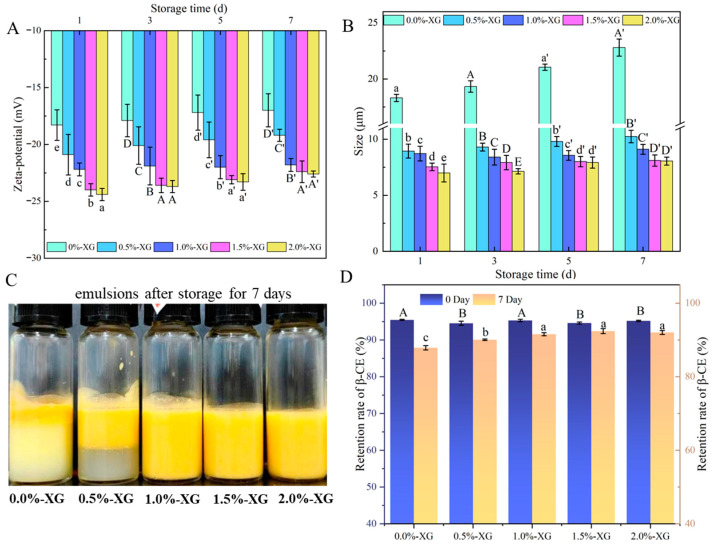
Effects of different XG concentrations on zeta potential (**A**), particle size (**B**), visual observations (**C**), and retention rate (**D**) of XG-SFOB-β-CE emulsions during 7 days of storage. Different lowercase and uppercase letters indicate significant differences (*p* < 0.05) for different XG concentrations on the same storage day.

To evaluate the effect of different XG concentrations on the stability of SFOB-β-CE emulsions during storage at 4 °C for one week, we monitored changes in zeta potential and particle size on days 0, 3, 5, and 7. The results in Figure 2 show that, over time, the absolute values of the zeta potential for all samples gradually decreased at nearly the same rate. However, the samples with 1.5% and 2.0% XG maintained the highest absolute zeta potential values throughout storage.

For particle size, although all samples showed an increase during storage, the 0% XG sample exhibited the largest increase, from 17.9 μm to 22.8 μm. As the XG concentration increased, particle size remained relatively stable, with the 1.5% and 2.0% XG samples maintaining smaller sizes throughout storage. This stability is mainly attributed to the higher surface charge of the coated SFOB-β-CE droplets, which results in stronger electrostatic repulsion within the emulsion, significantly reducing collisions between particles and thus improving system stability and preventing massive coalescence.

Figure 2C shows the texture of the XG-SFOB-β-CE emulsions after storage in a refrigerator at 4 °C in the dark for 7 days. From Figure 1B and Figure 2, it is clear that the emulsions remained almost unchanged after 7 days of storage. This behavior is most evident in samples containing 1.0%, 1.5%, and 2.0% XG, likely due to XG’s ability to increase the viscosity of the continuous phase [36], which hinders the movement of emulsion droplets. This prevents oil droplets from easily aggregating into larger clusters and reduces phase separation. The protective effect of XG on the encapsulated bioactive was quantitatively confirmed by measuring the β-CE retention rate after 7 days of storage (Figure 2D). All freshly prepared emulsions (Day 0) exhibited similarly high retention rates (−95%) with no significant differences. The emulsion without XG (0%) experienced the greatest loss of β-CE after 7 days of storage. In contrast, increasing the XG concentration progressively improved retention, with the 1.5% and 2.0% XG emulsions maintaining the highest final β-CE levels. This correlation clearly demonstrates that XG-induced stabilization, observed physically through higher zeta potential, smaller particle size growth, and visual homogeneity, leads to protection of the encapsulated lipophilic compound.

### 3.4. The Influence of Xanthan Gum on the Thermal Stability and Oxidative Stability of SFOB-β-CE Emulsion

**Figure 3:**
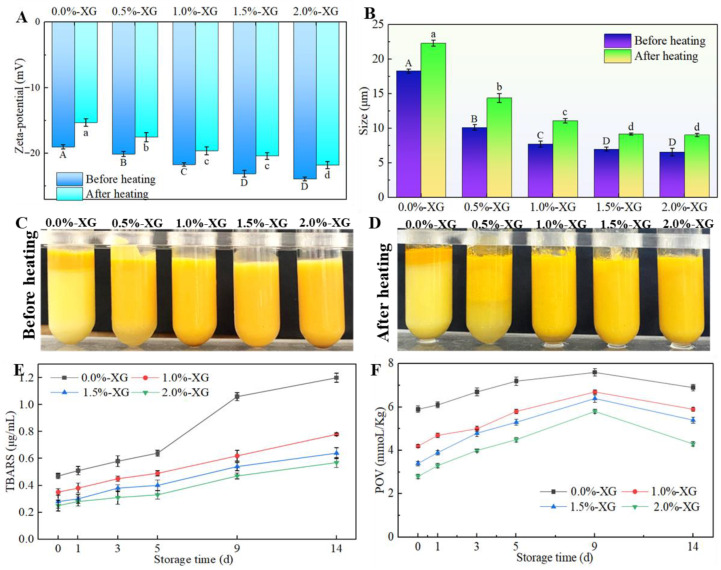
Effects of different XG concentrations on zeta potential (**A**), particle size (**B**), and visual observations (**C**,**D**) of non-heated and heated XG-SFOB-β-CE emulsions, as well as TBARS (**E**) and POV (**F**) of XG-SFOB-β-CE emulsions stored at 37 °C for 0–14 days. Different lowercase and uppercase letters indicate significant differences (*p* < 0.05) among the different XG concentrations.

Heat treatment is a common and important process in both the food and pharmaceutical industries. To evaluate the effectiveness of XG coating during thermal treatment, we again used four different XG concentrations. The samples were exposed to 100 °C for 15 min, and we examined their zeta potential, particle size, and visual observation. The results in Figure 3 showed that the absolute value of the zeta potential decreased in all samples. This decrease may be explained by the fact that heat treatment can cause structural relaxation of XG, which in turn reduces the density of negative charges exposed at the droplet interface [37].

After heat treatment, all emulsions showed an increase in particle size, but the extent of this increase varied depending on the presence and concentration of XG. The naked SFOB-β-CE emulsion (0% XG) exhibited the largest size increase, suggesting that without a protective layer, the droplets were more prone to collision and aggregation under thermal stress [28]. In contrast, coated samples had relatively smaller size increases, indicating that XG helped protect the emulsions by increasing the viscosity of the surrounding phase and creating a steric barrier that limited droplet movement [32]. As mentioned earlier, the absolute value of zeta potential decreased in all samples after heating, but the coated emulsions still maintained relatively high negative charges. This means that even after thermal exposure, the XG-coated droplets were better able to repel each other, reducing the possibility of coalescence. Together, the steric and electrostatic stabilization provided by XG contributed to the improved thermal stability of the emulsions compared to uncoated SFOB-β-CE.

The visual evaluation of the freshly prepared XG-SFOB-β-CE emulsions heated at 100 °C for 15 min is shown in Figure 3C. In emulsions with XG concentrations below 1.0% (*w*/*w*), a surface lumping effect appeared after heating, with visible precipitation of β-CE on the top layer. The water phase at the bottom became more pronounced, leading to clear phase separation and reduced emulsion stability. In contrast, emulsions containing more than 1.0% XG showed no visible lumping or layering. This was expected based on the zeta potential and particle size results discussed above. These results suggest that XG, when used at sufficient concentrations, can effectively enhance the thermal stability of the emulsion by preventing phase separation and maintaining uniformity.

Freshly prepared coated and uncoated XG-SFOB-β-CE emulsions were stored at 37 °C for 14 days to evaluate the level of protection against oxidation provided by XG. Figure 3E,F showed the development of POV as a marker of primary oxidation and TBARS as an indicator of secondary oxidation. The content of lipid hydroperoxides increased with storage time in all samples, reaching the highest level on day 9 before beginning to decline. This decline may be explained by the conversion of some primary oxidation products into secondary products [38].

Notably, the uncoated emulsion showed the highest POVs, and as the concentration of XG increased, POV values decreased. This suggests that XG can protect the lipid core of SFOBs against oxidation, likely by forming an impermeable coating around the droplets. A thicker XG layer provides stronger protection by creating a thicker interfacial coat at the oil-water interface. Furthermore, xanthan gum exhibits antioxidant activity by chelating pro-oxidative transition metals. For example, it was previously reported that XG binds Fe^2+^ ions through its pyruvate residues, thereby suppressing metal-catalyzed lipid peroxidation [39]. Other studies have also shown that adding XG can delay lipid oxidation in emulsions due to this combined mechanism of physical shielding and metal chelation [40,41,42]. Figure 3E showed that secondary oxidation followed a similar trend. TBARS values increased during storage in all emulsions, with the highest values observed in the uncoated emulsion. Increasing XG concentrations resulted in lower TBARS values, indicating that XG also inhibits the formation of secondary oxidation products. In conclusion, the addition of XG enhances the oxidative stability of SFOB-β-CE emulsions by providing both physical protection and a barrier effect that slows lipid oxidation.

### 3.5. Light Stability

**Figure 4:**
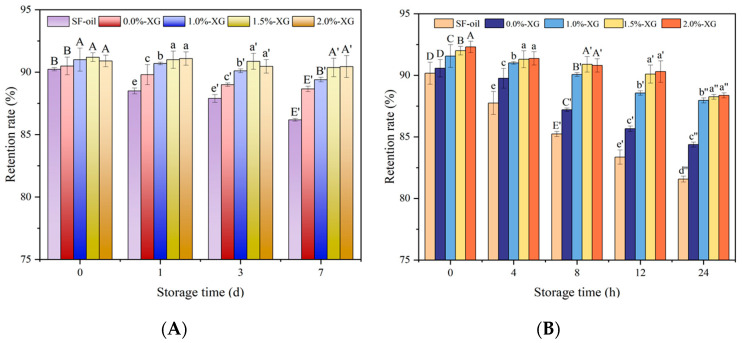
Effects of different XG concentrations on sunlight (**A**) and UV (**B**) stabilization of XG-SFOB-β-CE emulsions. Different lowercase and uppercase letters indicate significant differences (*p* < 0.05) among XG concentrations on the same storage day.

Due to the light sensitivity of β-carotene, we investigated its photostability in XG-SFOB emulsions under two light exposure conditions: simulated sunlight and UV irradiation. As storage time increased, the retention rate of β-CE in all samples gradually decreased. The retention rates of XG-SFOB-β-CE emulsions were higher than those of the control group (β-CE in sunflower oil). Figure 4A shows that during exposure to normal light, a decrease in β-CE retention was observed in all samples, with the level of degradation inversely related to the concentration of XG. After one day, emulsions with 0% XG and 1% XG already showed a noticeable decline in β-CE content, although it was not statistically significant (*p* < 0.05), whereas emulsions with 1.5% and 2% XG exhibited relatively stable retention. By day 7, all samples showed some degradation, but the 0% XG emulsion had the lowest retention rate, indicating poor protective capability. These findings suggest that XG forms a thicker interfacial layer that limits oxygen and light penetration.

Under UV irradiation (Figure 4B), the uncoated emulsion (0% XG) showed a sharp decrease in β-CE retention within the first 8 h, and retention continued to decline steeply with increased exposure time. Similar to the trend observed under normal light, emulsions with higher XG concentrations (particularly 1.5% and 2%) demonstrated significantly (*p* < 0.05) better resistance to UV degradation. This protective effect appears to depend on the concentration of XG, as lower XG levels (0% and 1%) provided limited stabilization, likely due to insufficient coating thickness.

These results confirm the crucial role of the XG layer in improving the photostability of β-CE in SFOB-β-CE-based emulsions. Thicker interfacial layers formed by higher concentrations of XG likely hindered the diffusion of reactive species and absorbed or scattered light more effectively, thereby reducing the rate of β-CE photodegradation. Our results also show that UV light causes more severe damage to β-CE stability compared to visible light, making the use of biopolymer coatings particularly important for light-sensitive compounds in food and pharmaceutical applications.

### 3.6. In Vitro Digestion of SFOB-β-CE Emulsions with Different XG Concentrations

#### 3.6.1. Tricine-SDS-PAGE Analysis

**Figure 5:**
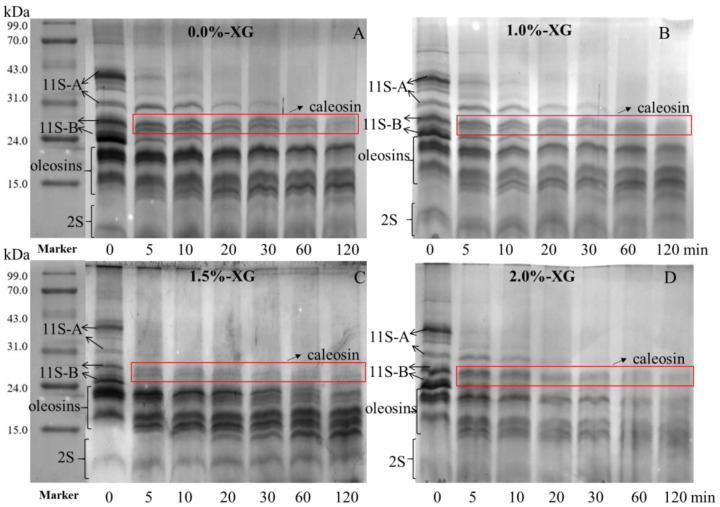
Tricine-SDS-PAGE of XG-SFOB-β-CE emulsion during gastric digestion. (**A**) 0.0%-XG; (**B**) 1.0%-XG; (**C**) 1.5%-XG; (**D**) 2.0%-XG.

The hydrolysis of uncoated and coated SFOB-β-CE emulsions by pepsin at the gastric digestion level was evaluated using reducing Tricine-SDS-PAGE (Figure 5). The gel showed the presence of oleosin, caleosin, and the extrinsic protein 11S globulin on the SFOB. The 11S globulin consisted of acidic (26–36 kDa) and basic (17–24 kDa) polypeptides [23]. Previous reports indicated that SFOBs contain three oleosins: 16, 17.5, and 20 kDa [43]. In Figure 5A, two distinct bands were observed between 17 and 23 kDa. The broad band around 18 kDa was actually two overlapping bands, which became very clear after 5 min of pepsin digestion in uncoated SFOB-β-CE and in all coated samples even before digestion began. Between the 22 and 27 kDa bands, two bands likely corresponding to another intrinsic protein, caleosin, were observed. These proteins are structural components that provide stability and integrity to SFOBs. Pepsin digestion showed that the uncoated sample displayed visible and well-defined protein bands throughout the digestion period, particularly low molecular weight oleosins (L-oleosin), indicating that surface proteins remained relatively resistant to gastric proteolysis. This is expected, as oil bodies naturally exhibit high structural stability, and L-oleosin specifically is always resistant to endogenous proteases compared to high molecular weight oleosins and caleosin [12].

On the other hand, we investigated the effect of XG and its concentration on pepsin digestion. Results in Figure 5B–D show that as XG content increases, the hydrolysis of SFOBs also increases. In particular, diffuse and faint bands were observed, especially after 60 and 120 min, indicating more extensive protein degradation and solubilization. This suggests that a thicker XG coating does not protect the protein interface; rather, it may make it more accessible to pepsin. The increased hydrolysis could also result from improved enzyme diffusion in the hydrated matrix surrounding the droplets or from interfacial restructuring caused by interactions between XG and the oil body surface. Lan et al. (2026) reported that 0.3% (*w*/*w*) XG affected the conformation of the surface proteins of safflower oil body and enhanced FFA release [44]. Overall, these results show that XG can enhance protein digestibility in a concentration-dependent manner, with 2% XG promoting the most extensive proteolysis.

#### 3.6.2. Zeta Potential, Particle Size, FFA Release and Bioaccessibility

**Figure 6:**
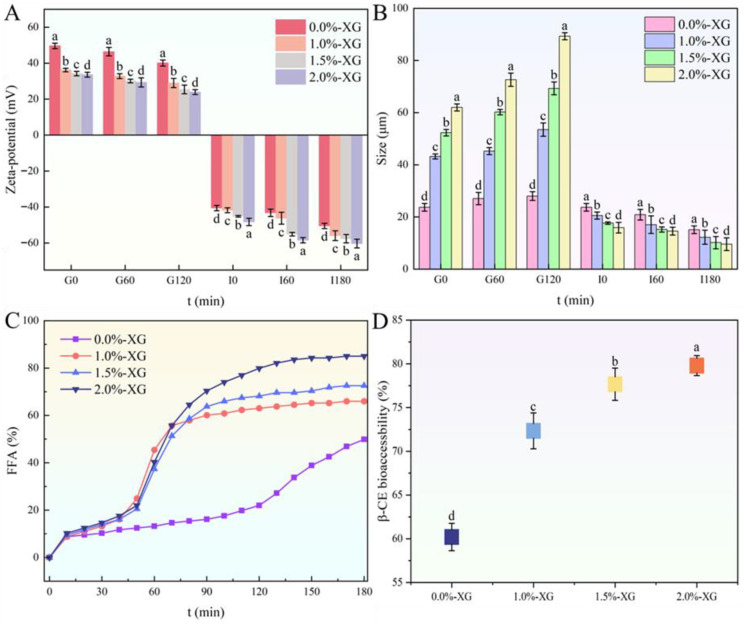
(**A**) Zeta potential, (**B**) average particle size, (**C**) free fatty acid release and (**D**) bioaccessibility of β-CE. Different lowercase letters indicate significant differences (*p* < 0.05).

As shown in Figure 6A, the zeta potential results showed a significant change at the interfaces during digestion. In the gastric phase, the uncoated sample showed the highest positive zeta value, probably due to protonation of the surface-exposed amino groups of the protein at low pH. This is because the surface of the oil body was fully exposed and unshielded. In contrast, the coated emulsions (1.0–2.0%) showed lower positive zeta values, indicating some degree of surface shielding. Since XG is negatively charged and highly hydrophilic, it probably masked the protein surface and altered the distribution of charges at the oil-water interface.

During intestinal digestion, all emulsions developed negative zeta potentials due to bile salt adsorption and FFA release. The 2% XG emulsion maintained the highest charge (−60 mV), reflecting enhanced bile binding and deprotonation of carboxyl groups on XG at neutral pH; -COOH groups convert to COO^−^, increasing the absolute value of the zeta potential. In contrast, the uncoated emulsion showed a lower charge (−40 mV), likely due to structural collapse earlier in the process, which limited the available surface area for bile and lipase interaction. These results demonstrate that XG coatings stabilize interfacial charge dynamics, directly mediating digestive efficiency.

The particle size data reflected important structural changes in the SFOB-β-CE emulsions during simulated digestion (Figure 6B). In the gastric phase, the size of all emulsions increased to varying degrees, but the most notable increase occurred in the sample coated with 2% XG, which exceeded 90 µm at the end of digestion, compared to around 27 µm for the uncoated samples. This increase in size at high XG concentration is likely due to aggregation resulting from weak steric repulsion caused by the low absolute value of the zeta potential. At low pH, the carboxyl groups of XG undergo protonation, reducing the negative charge on XG and leading to less electrostatic repulsion between droplets. The droplets may temporarily agglomerate or flocculate, resulting in an increase in apparent droplet size. In contrast, the uncoated droplets remained relatively well dispersed. Huang et al. also reported that emulsions stabilized by XG were significantly influenced by pH, with larger droplet sizes observed at pH 3.0 [45]. This was accompanied by a weaker zeta potential at acidic pH compared to pH 7.0, indicating reduced electrostatic repulsion and emulsion stability under acidic conditions [45]. In the intestinal phase, the trend reversed. The 2% XG sample showed the smallest particle size, while the uncoated sample had a larger droplet size. This smaller size may reflect the formation of mixed micelles and smaller digestion products, which is consistent with the high absolute zeta potential observed in this phase.

As shown in Figure 6C, the release of free fatty acids (FFA) during the intestinal phase revealed major differences in how the emulsions responded to digestion, depending on the XG concentration. All samples showed a slow FFA release during the first 45 min. However, after this initial phase, the coated emulsions (1.0–2.0% XG) began releasing FFAs more rapidly, while the uncoated sample (0%) continued to digest at a much slower rate.

This behavior is strongly supported by the Tricine-SDS-PAGE results discussed earlier. In coated emulsions, the intrinsic interfacial proteins, especially oleosins and caleosins, were more extensively hydrolyzed during the gastric phase. We propose that the XG coating alters the oil body interface, potentially by interacting with the surface proteins and causing slight conformational changes or by creating a local environment that concentrates pepsin near the droplet surface, thereby enhancing proteolytic efficiency. As a result, the oil body structure became partially disrupted, making the core TAGs more accessible to pancreatic lipase once digestion transitioned to the intestine. In contrast, the uncoated oil bodies maintained a more intact interfacial protein layer, particularly L-oleosin, which likely continued to act as a barrier during the early stages of intestinal digestion. This would explain the delayed lipolysis and late surge in FFA release observed in the 0% XG sample after 90 min, when structural breakdown finally allowed enzymes to reach the lipid core.

Among all samples, the 2% XG emulsion achieved the highest overall FFA release (85.75%), indicating that a thicker XG coating did not hinder digestion; in fact, it contributed to destabilizing the interfacial layer earlier in the process. This highlights an interesting role of XG, where it initially protects the droplets from industrial aggressions, but during digestion it facilitates pepsin access to intrinsic proteins, ultimately promoting more efficient lipid digestion in the intestine.

Figure 6D shows that the bioaccessibility of β-CE increased significantly with XG concentration, from 60.2% in the uncoated sample to nearly 80% in the 2% XG emulsion. These results align with the FFA release curve discussed above, suggesting that improved lipid digestion also enhances the release and solubilization of lipophilic bioactives like β-CE. The lower bioaccessibility in the uncoated emulsion is likely due to the slower breakdown of oil bodies, as the L-oleosin layer remained relatively intact and resisted digestion. Without efficient lipid hydrolysis, β-CE remains trapped inside the oil core and is less available for micellization and absorption. In contrast, emulsions coated with XG, particularly at 1.5% and 2%, showed greater protein degradation (as evidenced by Tricine-SDS-PAGE) and faster lipid digestion (via FFA release), which likely led to more effective β-CE release into the intestinal phase.

Overall, this confirms that XG not only stabilizes the emulsion system during processing and digestion but also significantly enhances the nutritional delivery of encapsulated bioactives such as β-CE.

### 3.7. Advantages, Limitations, and Potential Food Applications

This study demonstrates a practical method for stabilizing β-carotene using clean-label ingredients: sunflower oil bodies and xanthan gum. The main advantages of this approach are its use of natural components, the elimination of synthetic emulsifiers, and the effective protection of β-CE against physical separation, oxidation, and light-induced degradation. The improved bioaccessibility further indicates a potential nutritional benefit.

On the other hand, the primary limitations include the need for specialized equipment and conditions (ultrasonication at 270 W, homogenization at 80 MPa) and the extra step of OB extraction. The stability was also only verified over one week; performance in real food systems with different pH, salts, or ingredients requires future investigations.

For potential food applications, this emulsion system is suitable for fortifying liquid or semi-liquid products such as functional beverages, nutritional shakes, smoothies, salad dressings, and sauces, where its stability against light and oxidation would be particularly valuable.

## 4. Conclusions

This study demonstrates a systematic approach to improving the encapsulation efficiency, stability, and delivery of β-carotene (β-CE) using sunflower oil body (SFOB) emulsions. SFOB and β-CE were mixed at 45 °C for 15 min, then treated with 270 W ultrasonic for 20 min, followed by homogenization at 80 MPa, resulting in a β-CE encapsulation rate of up to 92%. To address limitations and improve stability under various environmental conditions and industrial stresses, we incorporated xanthan gum (XG). Results indicated that at concentrations above 1.5%, XG increased negative charges (zeta potential: −21.3 mV to −23.7 mV), reduced droplet size (6.52 μm), and improved storage and thermal stability (lower zeta potential and smaller size), photosensitivity (higher retention rate of β-CE), and oxidative stability (POV: 4.3 mmol/kg, TBARS: 0.57 μg/mL). Furthermore, during digestion, XG-coated emulsions showed increased protein hydrolysis and lipid breakdown, facilitating the release and micellization of β-CE. Notably, the 2% XG sample achieved the highest levels of free fatty acids (85.75%) and β-CE utilization (80%). However, limited polysaccharide regulated the stability and digestion behavior of SFOB-β-CE emulsions. Further research can focus on types of polysaccharides, in vivo validation, and sensory evaluation. These findings confirm that XG enhances the structural integrity and functional performance of SFOB-based delivery systems. This research underscores the feasibility of using natural oil bodies to encapsulate lipophilic nutrients and reveals the promising role of hydrocolloids such as XG in developing stable, functional emulsions for nutraceutical applications.

## Figures and Tables

**Table 1:** Effects of different treatments on EE, zeta potential and size of SFOB-β-CE emulsions during storage (0 and 7 days).

Treatment Conditions		Encapsulation Efficiency (%)		Zeta Potential (mV)		Size (μm)	
		0 days	7 days	0 days	7 days	0 days	7 days
Heating temperature	30 °C	45.6 ± 0.5 ^d^	-	−21.1 ± 0.2 ^c^	-	17.1 ± 0.2 ^b^	-
	35 °C	52.3 ± 0.7 ^c^	-	−21.4 ± 0.3 ^c^	-	17.2 ± 0.3 ^b^	-
	40 °C	89.4 ± 0.9 ^b^	84.9 ± 0.7 ^b^	−22.4 ± 0.4 ^b^	−18.7 ± 0.3 ^a^	17.5 ± 0.5 ^b^	25.2 ± 0.6 ^a^
	45 °C	92.1 ± 0.7 ^a^	88.5 ± 0.7 ^a^	−23.2 ± 0.4 ^a^	−18.8 ± 0.5 ^a^	18.2 ± 0.4 ^a^	25.3 ± 0.6 ^a^
Heating time	5 min	84.2 ± 0.2 ^c^	79.0 ± 0.5 ^a^	−24.1 ± 0.3 ^a^	−19.3 ± 0.4 ^a^	17.7 ± 0.3 ^d^	24.8 ± 0.5 ^c^
	15 min	91.9 ± 0.2 ^a^	87.8 ± 1.0 ^b^	−23.5 ± 0.2 ^b^	−18.6 ± 0.5 ^b^	18.8 ± 0.2 ^c^	30.0 ± 0.2 ^b^
	30 min	89.8 ± 0.6 ^b^	87.7 ± 0.5 ^b^	−22.7 ± 0.4 ^c^	−18.1 ± 0.1 ^b^	23.5 ± 0.4 ^b^	33.3 ± 0.3 ^a^
	45 min	90.4 ± 1.4 ^b^	86.7 ± 1.4 ^b^	−21.3 ± 0.2 ^d^	−17.9 ± 0.7 ^bc^	25.3 ± 0.2 ^a^	33.3 ± 0.4 ^a^
Ultrasonic power	90 W	81.2 ± 0.7 ^d^	73.8 ± 0.5 ^d^	−20.1 ± 0.2 ^bc^	−19.9 ± 0.1 ^b^	21.5 ± 0.2 ^a^	28.3 ± 0.3 ^a^
	150 W	83.8 ± 0.8 ^c^	76.0 ± 0.3 ^c^	−21.5 ± 0.2 ^b^	20.0 ± 0.1 ^b^	20.3 ± 0.2 ^ab^	27.5 ± 0.5 ^a^
	210 W	88.8 ± 0.8 ^b^	80.2 ± 0.5 ^b^	−21.5 ± 0.2 ^b^	−20.9 ± 0.1 ^a^	18.9 ± 0.3 ^c^	25.8 ± 0.3 ^b^
	270 W	92.5 ± 0.9 ^a^	87.9 ± 0.4 ^a^	−22.8 ± 0.3 ^a^	−21.1 ± 0.1 ^a^	17.8 ± 0.3 ^c^	20.0 ± 0.6 ^c^
	330 W	91.1 ± 0.5 ^a^	87.5 ± 0.2 ^a^	−22.2 ± 0.6 ^a^	−20.8 ± 0.2 ^a^	18.2 ± 0.4 ^c^	21.2 ± 0.4 ^c^
Ultrasonic time	10 min	83.9 ± 0.9 ^c^	80.8 ± 0.7 ^b^	−21.3 ± 0.3 ^c^	−17.1 ± 1.0 ^b^	26.0 ± 0.3 ^a^	39.6 ± 0.2 ^a^
	20 min	91.1 ± 0.4 ^a^	87.5 ± 1.6 ^a^	−23.5 ± 0.2 ^b^	−18.1 ± 0.6 ^a^	19.8 ± 0.2 ^b^	29.0 ± 0.1 ^b^
	40 min	89.8 ± 0.8 ^b^	87.0 ± 1.6 ^a^	−24.2 ± 0.1 ^a^	−18.6 ± 1.0 ^a^	18.1 ± 0.1 ^c^	22.3 ± 0.3 ^c^
	60 min	90.7 ± 0.6 ^b^	87.0 ± 0.7 ^a^	−24.6 ± 0.2 ^a^	−18.4 ± 0.8 ^a^	17.9 ± 0.2 ^c^	20.3 ± 0.4 ^d^
Homogenizing pressure	20 MPa	85.8 ± 0.6 ^c^	83.5 ± 0.5 ^c^	−19.5 ± 0.3 ^c^	−17.1 ± 0.6 ^b^	21.4 ± 0.3 ^a^	30.0 ± 0.8 ^a^
	50 MPa	87.5 ± 1.1 ^b^	85.5 ± 0.1 ^b^	−20.0 ± 0.2 ^c^	−17.3 ± 0.4 ^ab^	19.1 ± 0.3 ^b^	25.0 ± 0.1 ^b^
	80 MPa	90.8 ± 0.6 ^a^	87.8 ± 0.8 ^a^	−21.5 ± 0.13 ^b^	−18.3 ± 0.73 ^a^	17.7 ± 0.1 ^c^	24.2 ± 0.1 ^c^
	110 MPa	89.9 ± 0.5 ^a^	86.8 ± 0.4 ^a^	−22.7 ± 0.36 ^a^	−18.5 ± 0.61 ^a^	15.1 ± 0.4 ^d^	22.1 ± 0.2 ^d^

Values are means ± standard deviation. Different letters within the same column are significantly different at *p* < 0.05.

## Data Availability

The original contributions presented in this study are included in the article/Supplementary Materials. Further inquiries can be directed to the corresponding author.

## Supplementary Materials

The following supporting information can be downloaded at: https://www.mdpi.com/article/10.3390/foods15030567/s1, Figure S1. The schematic diagram illustrating the different emulsions preparation process. Figure S2. The standard curve of β-CE. Figure S3. The standard curve of Fe^3+^.

## References

[1] Sereti F., Alexandri M., Papapostolou H., Papadaki A., Kopsahelis N. (2025). Recent progress in carotenoid encapsulation: Effects on storage stability, bioaccessibility and bioavailability for advanced innovative food applications. Food Res. Int..

[2] Cavdar Dincturk H., Akkuzu N., Günal-Köroğlu D., Can Karaca A., Capanoglu E. (2026). Molecular Insights into Bioactive Interactions Within Protein- and Polysaccharide-Based Colloids: Implications for Stability, Functionality, and Bioavailability. Foods.

[3] Kasperczyk S., Dobrakowski M., Kasperczyk J., Ostałowska A., Zalejska-Fiolka J., Birkner E. (2014). Beta-carotene reduces oxidative stress, improves glutathione metabolism and modifies antioxidant defense systems in lead-exposed workers. Toxicol. Appl. Pharmacol..

[4] Gul K., Tak A., Singh A., Singh P., Yousuf B., Wani A.A. (2015). Chemistry, encapsulation, and health benefits of β-carotene-A review. Cogent Food Agric..

[5] Mankan E., Karakas C.Y., Saroglu O., Mzoughi M., Sagdic O., Karadag A. (2025). Food-Grade Liposome-Loaded Delivery Systems: Current Trends and Future Perspectives. Foods.

[6] Chen J., Li F., Li Z., McClements D.J., Xiao H. (2017). Encapsulation of carotenoids in emulsion-based delivery systems: Enhancement of β-carotene water-dispersibility and chemical stability. Food Hydrocoll..

[7] Liu W., Wang J., McClements D.J., Zou L. (2018). Encapsulation of β-carotene-loaded oil droplets in caseinate/alginate microparticles: Enhancement of carotenoid stability and bioaccessibility. J. Funct. Foods.

[8] Zhang Z., Zhang R., McClements D.J. (2016). Encapsulation of β-carotene in alginate-based hydrogel beads: Impact on physicochemical stability and bioaccessibility. Food Hydrocoll..

[9] Mahmood T., Akhtar N., Manickam S. (2014). Interfacial film stabilized W/O/W nano multiple emulsions loaded with green tea and lotus extracts: Systematic characterization of physicochemical properties and shelf-storage stability. J. Nanobiotechnology.

[10] McClements D.J., Bai L., Chung C. (2017). Recent advances in the utilization of natural emulsifiers to form and stabilize emulsions. Annu. Rev. Food Sci. Technol..

[11] Zhang S., Chen H., Geng F., Xie B., Sun Z., Huang Q., Peng D., Chen Y., Deng Q. (2023). Solvent-free encapsulation of β-carotene in natural flaxseed oil bodies induced via tepidity-physical field treatment: Formation, characteristic and stability. Food Hydrocoll..

[12] Zaaboul F., Zhao Q., Xu Y., Liu Y. (2022). Soybean oil bodies: A review on composition, properties, food applications, and future research aspects. Food Hydrocoll..

[13] Zhang S., McClements D.J., Zheng R., Yu X., Sun Z., Xie B., Chen Y., Deng Q. (2025). A promising perspective to boost the utilizability of oil bodies: Moderate regulation and modification of interface. Compr. Rev. Food Sci. Food Saf..

[14] Yang R., Deng H., Zhao Y., Lin H., Song Y., Zhao L., Miao W., Zheng B. (2025). Interface engineering of plant oil body for an innovative food ingredient: A review. Trends Food Sci. Technol..

[15] Zhang S., Chen H., Geng F., Peng D., Xie B., Sun Z., Chen Y., Deng Q. (2022). Natural oil bodies from typical oilseeds: Structural characterization and their potentials as natural delivery system for curcumin. Food Hydrocoll..

[16] Fisk I.D., White D.A., Carvalho A., Gray D.A. (2006). Tocopherol—An intrinsic component of sunflower seed oil bodies. J. Am. Oil Chem. Soc..

[17] Li Z., Zheng S., Zhao C., Liu M., Zhang Z., Xu W., Luo D., Shah B.R. (2020). Stability, microstructural and rheological properties of Pickering emulsion stabilized by xanthan gum/lysozyme nanoparticles coupled with xanthan gum. Int. J. Biol. Macromol..

[18] Zhu J., Liu L., Li X., Zhang Q., Wang Z., Chen N., Wang H., Xie F., Qi B., Jiang L. (2024). Construction of soybean oil bodies–xanthan gum composite oleogels by emulsion-templated method: Preparation, characterization, and stability analysis. Food Hydrocoll..

[19] Yamamoto Y., Sogawa I., Nishina A., Saeki S., Ichikawa N., Iibata S. (2000). Improved Hypolipidemic Effects of Xanthan Gum-Galactomannan Mixtures in Rats. Biosci. Biotechnol. Biochem..

[20] Nikiforidis C.V., Kiosseoglou V. (2009). Aqueous extraction of oil bodies from maize germ (*Zea mays*) and characterization of the resulting natural oil-in-water emulsion. J. Agric. Food Chem..

[21] Tian Y., Zhao X., Wang Z., Zhang W., Jiang Z. (2024). Structural characteristics and stability analysis of coconut oil body and its application for loading β-carotene. Food Chem..

[22] Shen P., Yang R., Wu Y., Liu J., Ding X., Wang W., Zhao L. (2023). Effects of *Quillaja Saponin* on Physicochemical Properties of Oil Bodies Recovered from Peony (*Paeonia ostii*) Seed Aqueous Extract at Different pH. Foods.

[23] Zhao L., Chen Y., Yan Z., Kong X., Hua Y. (2016). Physicochemical and rheological properties and oxidative stability of oil bodies recovered from soybean aqueous extract at different pHs. Food Hydrocoll..

[24] He S., Zhou S., Guo W., Wang Y., Liu C., Wang R., Xiao F. (2020). Investigation of curcumin emulsion stability and gastrointestinal digestion prepared with rapeseed oil body. J. Food Process Eng..

[25] Sun Y., Zhong M., Wu L., Wang Q., Li Y., Qi B. (2022). Loading natural emulsions with nutraceuticals by ultrasonication: Formation and digestion properties of curcumin-loaded soybean oil bodies. Food Hydrocoll..

[26] Fu D., Deng S., McClements D.J., Zhou L., Zou L., Yi J., Liu C., Liu W. (2019). Encapsulation of β-carotene in wheat gluten nanoparticle-xanthan gum-stabilized Pickering emulsions: Enhancement of carotenoid stability and bioaccessibility. Food Hydrocoll..

[27] Gao Y., Zheng Y., Yao F., Chen F. (2022). Effects of pH and temperature on the stability of peanut oil bodies: New insights for embedding active ingredients. Colloids Surf. A Physicochem. Eng. Asp..

[28] Zaaboul F., Matabaro E., Raza H., Xin B.D., Duhoranimana E., Cao C., Liu Y. (2018). Validation of a simple extraction method for oil bodies isolated from peanuts. Eur. J. Lipid Sci. Technol..

[29] Cai Y., Deng X., Liu T., Zhao M., Zhao Q., Chen S. (2018). Effect of xanthan gum on walnut protein/xanthan gum mixtures, interfacial adsorption, and emulsion properties. Food Hydrocoll..

[30] Hemar Y., Tamehana M., Munro P.A., Singh H. (2001). Influence of xanthan gum on the formation and stability of sodium caseinate oil-in-water emulsions. Food Hydrocoll..

[31] Zhang J., Li G., Xu D., Cao Y. (2022). Enhancing the dispersion stability and sustained release of S/O/W emulsions by encapsulation of CaCO3 droplets in sodium caseinate/xanthan gum microparticles. Foods.

[32] Xu W., Li Z., Sun H., Zheng S., Li H., Luo D., Li Y., Wang M., Wang Y. (2022). High internal-phase pickering emulsions stabilized by xanthan gum/lysozyme nanoparticles: Rheological and microstructural perspective. Front. Nutr..

[33] Dickinson E. (2009). Hydrocolloids as emulsifiers and emulsion stabilizers. Food Hydrocoll..

[34] Manoi K., Rizvi S.S.H. (2009). Emulsification mechanisms and characterizations of cold, gel-like emulsions produced from texturized whey protein concentrate. Food Hydrocoll..

[35] Yang R., Juma N.S., Zhao Y., Zheng B., Xu Y., Gao Y., Jia R., Gao P., He Y. (2025). Factors influencing surimi gelling properties and natural additive–based gel fortification strategies: A review. Compr. Rev. Food Sci. Food Saf..

[36] Krstonošić V., Pavlović N., Nikolić I., Milutinov J., Ćirin D. (2024). Physicochemical properties and stability of oil-in-water emulsions stabilized by soy protein isolate and xanthan gum. Int. J. Biol. Macromol..

[37] Bielska P., Cais-Sokolińska D., Dwiecki K. (2022). Effects of heat treatment duration on the electrical properties, texture and color of polymerized whey protein. Molecules.

[38] Wazir H., Chay S.Y., Zarei M., Hussin F.S., Mustapha N.A., Wan Ibadullah W.Z., Saari N. (2019). Effects of storage time and temperature on lipid oxidation and protein co-oxidation of low-moisture shredded meat products. Antioxidants.

[39] Shimada K., Muta H., Nakamura Y., Okada H., Matsuo K., Yoshioka S., Matsudaira T., Nakamura T. (1994). Iron-binding property and antioxidative activity of xanthan on the autoxidation of soybean oil in emulsion. J. Agric. Food Chem..

[40] Sun C., Gunasekaran S. (2009). Effects of protein concentration and oil-phase volume fraction on the stability and rheology of menhaden oil-in-water emulsions stabilized by whey protein isolate with xanthan gum. Food Hydrocoll..

[41] Sun C., Gunasekaran S., Richards M.P. (2007). Effect of xanthan gum on physicochemical properties of whey protein isolate stabilized oil-in-water emulsions. Food Hydrocoll..

[42] Sun C., Liang B., Sheng H., Wang R., Zhao J., Zhang Z., Zhang M. (2018). Influence of initial protein structures and xanthan gum on the oxidative stability of O/W emulsions stabilized by whey protein. Int. J. Biol. Macromol..

[43] Vandana S., Bhatla S.C. (2006). Evidence for the probable oil body association of a thiol-protease, leading to oleosin degradation in sunflower seedling cotyledons. Plant Physiol. Biochem..

[44] Lan X., Tao C., Zhang L., Guo J., Wu Z., Li J., Wang D., Wei J., Li X., Yang J. (2026). Safflower oil body emulsion digestion behavior and regulation of intestinal microorganisms: The influence of polysaccharide xanthan gum. Food Chem. X.

[45] Huang H., Tian Y., Bai X., Cao Y., Fu Z. (2023). Influence of the emulsifier sodium caseinate–xanthan gum complex on emulsions: Stability and digestive properties. Molecules.

