# Supplementary material for: Xanthan Gum-Stabilized Sunflower Oil Body Emulsions for β-Carotene Delivery: Preparation, Stability, and Digestion Behavior
Source: Foods. 2026 Feb 5;15(3):567. doi: 10.3390/foods15030567 (PMC12896790; doi:10.3390/foods15030567)
Supplement: Supplementary file 1 [file foods-15-00567-s001.zip › foods-4092048-supplementary.pdf]

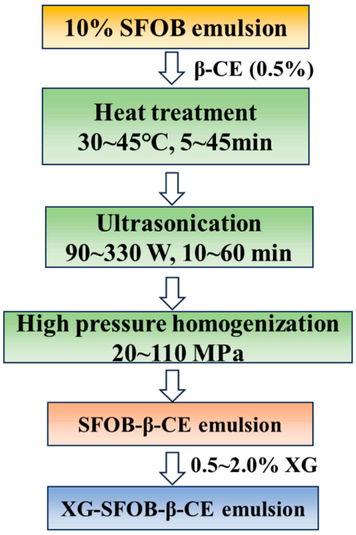

Figure S1. The schematic diagram illustrating the different emulsions preparation process.

The absorbance of the n-hexane/ethanol (2/1, v/v) solutions containing β-CE (0, 1, 2, 3, 4, 5 μg/mL) was measured at 450 nm. The n-hexane/ethanol (2/1,v/v) solution was used as the blank to prepare the standard curve (Figure S2).

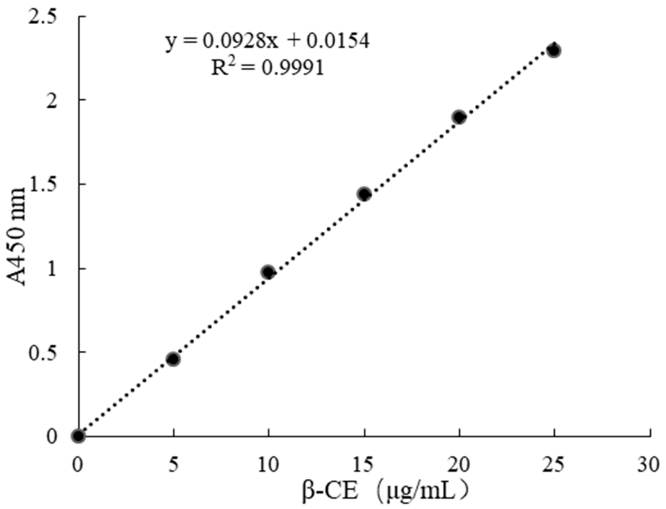

Figure S2. The standard curve of β-CE

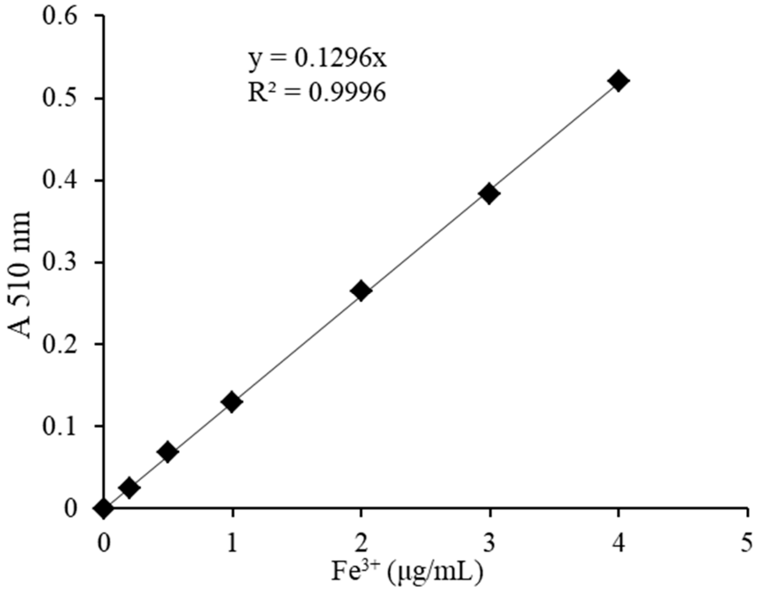

Figure S3. The standard curve of Fe^{3+}
